# Flirting with resistance: children’s expressions of autonomy during middle childhood

**DOI:** 10.1080/17482631.2018.1564519

**Published:** 2019-01-29

**Authors:** Leon Kuczynski, Robyn Pitman, Kate Twigger

**Affiliations:** a Department of Family Relations and Applied Nutrition, University of Guelph, Guelph, ON, Canada; b Department of Sociology, The University of British Columbia, Vancouver, BC, Canada

**Keywords:** Autonomy, child agency, non-compliance, resistance, socialization, parent–child relationships

## Abstract

**Purpose**: Developmental research suggests that children’s early non-compliance can be understood as “resistance”, an agentic response to parental control where children express their autonomy within a close relationship context. Research with toddlers and adolescents suggests that children’s resistance strategies can be differentiated using the dimensions of assertiveness, social skill, and overt versus covert expression. This study explores children’s strategies for expressing resistance during the neglected period of middle childhood.

**Method**: Forty children, 9–13 years of age, participated for 1 week in a study focused on children’s experiences of socialization and parent–child relationships. Procedures included a 5-day event diary, and a 1-hour semi-structured interview about the rules and expectations in their home and their strategies of resistance.

**Results**: Thematic analysis identified a rich repertoire of strategies for resisting unwelcome parental demands. These included overt resistance, such as negotiation, argument, and expressions of non-acceptance and covert resistance such as covert transgressions and cognitive non-acceptance of parental demands when compelled to comply.

**Conclusion**: The findings were interpreted as reflecting children’s development of assertiveness and social skill as they expressed their autonomy in the interpersonal context of the interdependent but asymmetrical relationship with their parents.

## Introduction

The words “non-compliance” and “disobedience”, when referring to children’s responses to parental requests, are usually associated with negativity, problem behaviour, and deviance. These meanings originate in cultural norms regarding children’s obedience to family and submission to parental authority (Trommsdorff & Kornadt, 2003) and idealized conceptions of parental firm control in the parenting literature (Baumrind, 2012; McMahon & Forehand, 2005). A repercussion of such conceptions of children’s non-conforming responses to parental expectations is that children’s attempts to resist restrictions on their choices are viewed as coercive and toxic to the well-being of both the parent and the child (Patterson, Reid, & Dishion, 1992).

A contrasting perspective is that children’s non-compliance with parental demands can be understood as “resistance”, a legitimate expression of their autonomy in the parent–child relationship (Kuczynski & Hildebrandt, 1997). Historically, the idea that non-compliance is an expression of autonomy can be traced to research during the 1920s on the phenomenon of “toddler negativism” or the “terrible twos”, which stemmed from the practical problem of understanding children’s refusal to answer items on early intelligence tests (Wenar, 1982). The emergence of “no” at around 18–24 months was interpreted as a manifestation of children’s attempts to assert themselves as individuals with needs that are separate from those of their parents. This idea was subsequently followed up in observational studies of children’s resistance to parents in toddler and preschool children (Crockenberg & Litman, 1990; Kuczynski & Kochanska, 1990; Kuczynski, Kochanska, Radke-Yarrow, & Girnius-Brown, 1987). Essentially, the argument is that childhood resistance is no different from adult assertion and is a manifestation of a universal motive of humans for self-determination and to protect freedom of action and choice. Parallels can be made between childhood resistance and adult concepts such as reactance to control (Brehm, 1981), self-determination (Deci & Ryan, 2000), and resistance to oppression (Goffman, 1961; Scott, 1990; Wade, 1997).

Viewing children’s non-conformity through an agentic lens is important for children’s health and well-being because it challenges long-standing clinical perspectives that children’s failure to comply immediately and exactly with parental requests is invariably problematic and in need of suppression (McMahon & Forehand, 2005). Research suggests that only socially unskilful or dysregulated forms of resistance are related to negative outcomes in childhood (Crockenberg & Litman, 1990; Kuczynski & Kochanska, 1990; Kuczynski et al., 1987; Van Petegem, Vansteenkiste, Soenens, Beyers, & Aelterman, 2015). In addition, parental acquiescence to children’s resistance when it is appropriately expressed may support children’s developing autonomy (Kochanska & Kuczynski, 1991). Beneficial developmental outcomes such as better adjustment, achievement in school, better self-regulation, and fewer behavioural problems have been linked to parental autonomy support (Grolnick, Kurowski, Dunlap, & Hevey, 2000). Conversely, autonomy-suppressing or controlling parenting has been found to decrease overall well-being and increase problem behaviour (van der Kapp-Deeder, Vansteenkiste, Soenens, & Mabbe, 2017).

Observational research with children between the ages of 2 and 5 years has found that young children use a variety of strategies for expressing resistance, including passive non-compliance, simple refusal, negotiation, and defiance (Kuczynski & Kochanska, 1990; Kuczynski et al., 1987). These studies suggest that although motives for autonomy remain constant, the way that children express their agency through resistance in social interactions changes with age. First, children become more assertive in expressing their autonomy to parents as they develop. This is evidenced by decreases in passive forms of resistance such as ignoring the parent’s request and increases in confrontational strategies such as direct refusal and attempts to negotiate. Secondly, young children become more socially skilled in expressing their resistance as they age. For example, socially aversive forms of resistance, such as ignoring and refusing in an angry defiant manner, have been found to decrease between the ages of 2 and 5 years, whereas simple polite refusal and verbal negotiation increased during this period. Negotiation, in particular, is an example of a skilful strategy because it represents attempts by children to accommodate parental wishes or persuade parents to modify their demands while still pursuing their own goals (Kuczynski & Hildebrandt, 1997).

A third developmental trend comes from studies that examined the resistance strategies of 13–18-year-old adolescents. Consistent with previous research (Smetana & Asquith, 1994), Parkin and Kuczynski (2012) found that adolescents were confident in asserting the boundaries of parental authority. In addition, the study documents the further development of adolescents’ strategies for expressing resistance, such as negotiation strategies, assertive confrontational strategies, and a previously undocumented category of covert resistance, including covert transgressions, minimal compliance, and subversive challenges to parental authority. The large increase in sophistication in children’s ways of expressing resistance from toddlerhood to adolescence suggests that major changes had occurred during the intervening unexplored period of middle childhood.

Various factors during middle childhood have implications for children’s expression of agency through resistance. For example, in comparison to the preschool period, middle childhood is a period of rapid change in children’s cognitive capacities, and associated verbal and social skills (Collins & Madsen, 2003). Consistent with this, Sorbring (2009) found that 8-year-olds could readily describe their own and their parents’ perspectives during conflict situations and could report on their strategies for negotiating changes or avoiding confrontation while meeting their goals. In addition, important contextual changes such as spending more time outside the home, at school, and with peers means that children are outside the direct control of parents and have more opportunities for covert resistance. However, in comparison to adolescence, school-age children are cognitively and socially less mature and are more closely supervised by parents. Thus, middle childhood can be viewed as a transitional period when battles for independence and personal freedoms are still being negotiated as children push back on the reach of parental authority (Smetana, 2011).

The purpose of this study was to explore the phenomenon of children’s resistance strategies in middle childhood. We were interested in identifying children’s repertories of strategies for resisting parental control in a way that fills in the knowledge gap between early childhood and adolescence. Based on previous research, we were interested in children’s strategies for expressing resistance overtly during social interactions with parents, their strategies for resisting parental expectations covertly in the parent’s absence, and their perspectives about how and why they resist parental expectations and requests.

Conceptually, the current study was informed by social relational theory (Kuczynski, 2003; Kuczynski & Del Mol, 2015). Social relational theory provides a comprehensive model of what it means to be an agent that encompasses cognition, action, and motivation (Kuczynski, 2003). This means that analyses of children’s resistance may usefully extend to different modalities including children’s interpretive activities and strategic goal-oriented actions and emotions. In addition, a tenet of social relational theory is that children’s agency, influence, and power must be understood in the context of specific interpersonal relationships. Children’s experience of agency and their ability to influence their parents is enabled and constrained by the distinctive social context of a long-term, interdependent, asymmetrical, parent–child relationship. The power difference between parents and children is conceptualized relationally as an “interdependent asymmetry”, which means that power dynamics and influence between parents and children is dialectical in nature (Kuczynski & Del Mol, 2015). Thus, in contrast to relationships between unfamiliar adults and children, parents may be both vulnerable and receptive to their children’s influence and may offer considerable leeway for children’s expression of agency (Kuczynski, 2003).

## Method

The participants were 40 English-speaking children who were recruited from a medium-sized city in Southwestern Ontario, Canada. The participants were attending elementary school and ranged from 9 to 13 years of age. Twenty children (10 males, 10 females) were between the ages of 9 and 11 years and 20 children (10 males, 10 females) were 12 or 13 years old. Participants were predominantly Canadian or European. Several of the participants’ parents identified themselves as Metis, West Indian, and African.

This research was a component of the Socialization in Middle Childhood Study and was approved by the university research ethics board (protocol 06FE028). Families were given two $25 gift cards for their participation in the study. Children participated with their mothers in their homes during three phases occurring within a 1-week period. Although parallel data collection procedures were also used for the mothers, the present study focuses on children’s responses only.

Phase one was designed to introduce parents and children to the study, build rapport, and provide training to children about how to use the digital recorders for completing the Kids’ Daily Report (KDR). Building rapport was important because parents and children were asked to report separately and in private on sensitive incidents involving non-compliance and rule transgressions. Phase two consisted of the KDR which guided children to track and report on specified incidents regarding parent–child interactions and the parent–child relationship each day for 5 consecutive days. Daily digital diaries are used to understand ongoing contextualized experiences in everyday situations by minimizing the amount of time elapsed between an experience and the account of the experience (Bolger, Davis, & Rafaeli, 2003).  Three of the daily incident reports were relevant to this study: parental prohibitions, parental requests and prohibitions that the child disagreed with or resisted, and children’s behaviours that the parent did not know about. Additional probes asked the child to describe what happened in each incident and the child’s thoughts about them. The purpose of the diary method was primarily to obtain a descriptive representation of the children’s repertoires of resistance strategies based on their lived daily experiences.

During phase three, children participated in a 1-hour semi-structured interview which capitalized on the rapport and insights generated during the 5-day diary. The interview covered four broad topics: children’s views of parental rules and expectations, children’s views of their own resistance to parental expectations and requests, behaviour away from home and with peers, and parent–child intimacy. The goal of asking open-ended questions was to gain a more in-depth understanding of children’s meanings, motives, and intentions of the events reported during the diary experiences, as well as their views of parental expectations and practices in general. In practice, the digital diaries and open-ended interviews overlapped but provided complementary information.

### Thematic analysis

The interviews and daily digital diary reports were transcribed from audio recordings. Themes were identified using the procedures for theoretical thematic analysis described by Braun and Clarke (2006). The steps of thematic analysis included familiarization with data through repeated reading of the transcripts, creating initial categories, searching for overarching themes, evaluating themes, and labelling and conceptualizing themes. In the present study, the initial interpretation of the data was sensitized by existing linear and transactional perspectives on socialization and parental practices. However, throughout the analyses the researchers were alerted to novel ideas expressed by the participants that were not present in the literature.

Constant comparison (Chamraz, 2003) was used to continually assess the similarities and differences between coded segments and themes and between the emerging themes themselves. In qualitative research, the requirements of validity and reliability are met by the criterion of trustworthiness. Stiles (1993) suggested that a trustworthy study is one where the researcher’s theoretical orientation is outlined and intensively engages with the data, and the findings and emerging themes are confirmed by discussions with independent researchers during the analytical process. Throughout the analysis, coding was completed by all three authors, who met regularly to review the themes, discuss alternative interpretations, and ensure rigour in the constant comparison process. Analyses were aided by a qualitative data analysis software program, MAXQDA, to ensure the systematic categorization of data, documentation of the analytical process in memos, and interpretive comments assigned to narratives and codes.

## Results

All children in our middle childhood sample expressed their agency by resisting parental requests that they perceived as unwelcome, inconvenient, or counter to their beliefs. Our interpretations of children’s narratives indicated that children expressed their resistance in two ways: overtly and covertly. Overt resistance consisted of various ways that children opposed their parents during social interactions. Covert resistance consisted of expressions of resistance that were private or concealed from parents. An overview of the themes and subthemes revealed in this study can be found in Table I. The interpretations of these themes focus on children’s developing assertiveness and skill as agents in the interpersonal context of an interdependent but asymmetrical parent–child relationship.

**Table I.** Number of children describing specific strategies for expressing resistance.

Resistance strategy	Total *n*	Males *n*	Females *n*
Overt resistance strategies			
Unassertive resistance strategies			
Deflecting parental requests	20	8	12
Minimal compliance with parental requests	12	5	7
Assertive resistance strategies			
Negotiation	21	8	13
Arguing	13	6	7
Expressive non-acceptance	18	9	9
Covert resistance strategies			
Covert transgressions	33	17	16
Cognitive non-acceptance			
Conflicting goals	31	15	16
Conflicting perspectives	18	8	10

### Overt resistance

We identified five strategies that children used to express resistance overtly during parent–child interactions. Two strategies were considered to be relatively unassertive and the other three were considered to be relatively assertive as interpersonal influence strategies.

#### Unassertive resistance strategies

Unassertive resistance consisted of strategies whereby children resisted without directly communicating their opposition to the request. Instead, children resisted in a safe and unobtrusive manner. Unassertive resistance strategies are represented by the subthemes of *deflecting parental requests and minimal compliance with parental requests*.

#### Deflecting parental requests

Children reported that they avoided complying with parental requests by the tactic of not acknowledging or minimally acknowledging that the parent had made a request. For example, one girl described how she ignored her father’s request to do a household chore: “I pretended I didn’t hear him” (13-year-old female, Family 7); and a boy reported “I usually just don’t do it and I’ll wait for her to ask me again” (13-year-old male, Family 22). Another child said that she evaded compliance by sneaking out of the room when asked to do a chore: “I have a lot of people in my family. I just try and leave and get out of it. I leave and go into my bedroom and hopefully someone else will do it so I don’t have to” (13-year-old female, Family 26). It was apparent that children often regarded ignoring parental requests as a symbolic gesture that they pursued with little expectation of success.

Children also said that they attempted to deflect parents’ requests by stalling with an excuse or by minimally acknowledging that they heard the request without committing to follow through. For example, one girl exultantly reported that she acknowledged her mother’s request while putting her off with excuses: “I was like, ‘Okay mum, whatever, I have got to do my homework right now.’ So I kept just saying that I had to do my homework and then I didn’t ever really do my chores” (13-year-old female, Family 14). Other children reported that they communicated their willingness to comply with a parental request but avoided following through with insincere negotiations regarding the time frame for completion. For example, an 11-year-old boy discussed his well-practised script of stalling with promises of deferred cooperation:

My mom, like sometimes when I’m watching TV or something will say “come and eat dinner” and I’ll just say “one second”, and I’ll keep saying “one second” or “one minute” until my show is like five minutes over and she’ll say “come and eat now” and I’ll say “there’s only five minutes left” and she’ll say “fine finish the show” and then and after the show is done I’ll go eat. (11-year-old male, Family 13)

These deflections were interpreted as being unassertive from an interpersonal standpoint because children did not directly communicate or confront parents with their resistance. However, it seemed apparent that deflecting tactics was more skilful as an interpersonal strategy than ignoring, and were less likely to elicit enforcement by parents.

#### Minimal compliance with parental requests

Minimal compliance described children’s reports that they complied with a parental request in a resistant way that followed the “letter of the law” but not the “spirit of the law”. Rather than confronting parents with their opposition, children complied at the surface level by doing the bare minimum of what was asked of them. Examples of minimal compliance were reported for parental requests for cleaning, putting clothes away, doing chores, caring for pets, practising music, and spending time with parents. In response to a request to vacuum a floor, a 13-year-old boy (Family 11) said: “I didn’t really want to vacuum the living room and I did try and get out of it. What I did was I didn’t really vacuum the whole place, I kind of just vacuumed the highest tracked areas”. In this example, the child complied in a way that creatively incorporated his resistance.

Another child maintained a pretence of compliance while at the same time attempting to adhere to a self-chosen, albeit less exacting standard of neatness, that differed from that of his mother: “I can clean my room in like 15 minutes when it’s like super messy …. But it’s usually as clean as she wants it … so I usually just throw it into the closet or under the bed” (11-year-old male, Family 30). Another child took a similar approach to cleaning: “I just kind of get the spots that are noticeable” (12-year-old male, Family 10). In these examples, children engaged in small acts of resistance while making a show of compliance with their mother’s request. Children acquiesced to parental authority symbolically while still maintaining a sense of agency and control over how the requirement was carried out.

#### Assertive resistance strategies

Children also reported expressing resistance openly, without concealing their complete or partial disagreement with parental expectations. These strategies were considered to be assertive in that children directly confronted a more powerful parent with their disagreement. However, subthemes in this category, including *negotiation*, *arguing*, and *expressive non-acceptance*, indicated that children’s assertive strategies differed in their skill as social strategies for persuading parents to drop or modify their requests.

#### Negotiation

Negotiation consisted of children’s attempts to have the requirements of a parental rule or request changed by presenting persuasive rationales, asking parents for justifications, and proposing alternatives. Negotiation was distinct from other forms of overt resistance because children actively engaged parents in the attempt to achieve a mutually acceptable accommodation while at the same time pursuing their own goals. Most reports of negotiation were initiated by children who resisted some aspect of the parent’s requests, such as timing, quantity, or quality of compliance.

There were many reports by children that they attempted to negotiate requests so that they were apportioned more fairly among siblings. A 13-year-old female provides the following rationale for making chores more equal with her siblings:

But if it’s one person gets to clean to two bathrooms and clean their room and do the laundry, whereas I got to clean this room, the two rooms downstairs, the kitchen, and walk the dog—those are really big things. Whereas laundry is like the only big one on the other list. Whereas if I said “why not we split up the laundry, we’ll split up my rooms and I’ll split up hers and I don’t mind walking the dog because that’s fun but we need to fix this so that’s it’s more equal“. (Family 18)

Some reports indicated that negotiation took the form of an interactive process between parents and children, who worked together to find common ground. In some instances, parents apparently started the process as a way to scaffold the child’s cooperation. A 13-year-old female stated the following about a compromise that her mother initiated:

I didn’t want to take my tack and my saddle downstairs into the basement because it was really heavy … my mom said that if I brought it to the top of the stairs she would take it down the rest of the way … I did end up doing what my mom said. (Family 16)

In this example, the girl recognized her mother’s participation in the give and take of a negotiated transaction where there was mutual accommodation as both mother and child strove to meet their own goals. Other instances were less skilful and part of an uncooperative strategy of avoidance. One female described negotiating the timing of compliance after her first attempt of blatant refusal was resisted by her mother: “She asked me to clean up my toys in her bedroom and I said ‘no’ and I did try to get out of it by saying ‘can I do it later?’ and she said ‘yes’” (9-year-old female, Family 41).

Some children expressed confidence in their capacities for negotiating desirable outcomes. A 13-year-old female stated the following knowledge about her bargaining skills:

I tend to be really persuasive …. Or just say this dress I really want. I’m just saying all the good things that would happen if I wore it, like if I went to a wedding I could wear it there because it’s nice and formal too. And it’s nice and light and summery so I could also wear it to school if I put [on] a jacket. (Family 18)

This example exemplified assertive and skilful resistance, whereby the child made her wishes clear and provided a persuasive explanation that could lead to a mutually satisfying outcome.

#### Arguing

Many children said that they used the tactic of arguing with their parents as a way to express opinions or challenge requests with which they did not agree. In contrast to negotiation, arguing consisted of asserting to parents what they were prepared to do rather than asking for compromise or collaboration. Arguing also highlighted children’s attempts to maintain their power in a relationship context that is inherently asymmetrical. In these incidents, yelling and negative emotions were sometimes expressed by both children and parents in mutual exchanges of power assertion. A 9-year-old male (Family 37) discussed the pattern of arguing and yelling in his parent–child relationship:

Interview: Why do you think you start the argument?

Child: They ask me to do something and I say, “No”. They ask me again, “No”.

Interviewer: Okay. And then they start to get a little frustrated?

Child: Yeah.

Interview: Okay, and that’s when the yelling might start?

Child: Yeah.

A 13-year-old girl described how she yelled at her parents over an argument about her right to wear a specific outfit:

I start yelling back and be like “you know, S [sibling] wore this in grade 7 and I’m in grade 6 and I’m wearing it as a beach thing. She wore these everyday at school. You can’t get mad at me for it, it’s for beach! No one’s going to see anything anyways, oh my gosh”. (Family 18)

This child’s expression of resistance is noteworthy as a strategy that was assertive but unskilful, because the child’s arguments were angry and coercive.

From an interpersonal standpoint, the strategy of arguing is an assertive but unskilful display of agency. By arguing, children assertively communicated their opposition to the parents’ request or message. But as an interpersonal influence strategy, arguing is unskilful because displays of anger to coerce parents are an affront to the agency of parents, who may resist in turn.

However, it was apparent that, sometimes, children deliberately used arguing not to achieve an instrumental goal, but to pursue a relational goal of challenging the parents’ use of power in the relationship. For example, a 12-year-old male described his antagonistic approach to argument and the power struggle that it occasioned. “I try to come back with better reasons for me to do it and then have my way and … I just dish back what my parents dished to me … They disagree back so there is usually a long argument” (Family 10). Here, the apparent goal was not to persuade parents but to communicate opposition to unilateral parental authority in the parent–child relationship.

#### Expressive non-acceptance

Half of the participants indicated that they communicated resistance with non-verbal expressions of disagreement or challenges to parental authority. Children reported that they expressed resistance using tone of voice, body language, and dismissive gestures during exchanges with parents about requests, after reprimands, or during negotiations and arguments. Examples included slamming doors or stomping: “He got to pick last year so I really think it should be me so I stomped to my room” (8-year-old female, Family 9); eye rolling: “I’ll probably just go do it but as I’m doing it maybe roll my eyes a bit” (11-year-old female, Family 1); crying: “I asked my mom and my mom said no and I started to cry” (11-year-old female, Family 3); and resistant facial expressions: “They kind of sometimes restart the argument a bit but kind of let it drop after and I just sit down and give them dirty looks” (12-year-old male, Family 10). Despite being made to comply, children expressed their continuing resistance to parental authority with indirect gestures of non-acceptance.

It is important to note that children’s non-verbal expressions were more than coercive expressions of defiance. They also communicated what they perceived as genuine experiences of angry or hurt emotions. Examples of emotional experiences included irritation: “I was just telling my mum that I was getting irritated when she did that” (12-year-old female, Family 34); anger: “That gets really annoying and … I was sort of really mad” (11-year-old female, Family 3); and hurt: “This is very unfair and makes me feel hurt and very sad on the inside” (13-year-old male, Family 6). These examples demonstrate children’s expressions of autonomy and agency through non-verbal presentations. Children used body language, tone of voice, and emotions to protest against what they perceived as being unfair, unjust, and something they did not agree with.

### Covert resistance

Children also provided insight into a covert dimension of agency where resistance was enacted privately and with diminished possibility of parental detection. Two forms of covert resistance are represented in the subthemes of *covert transgressions*, which occurred outside the parent’s surveillance, and *cognitive non-acceptance*, which occurred in the child’s mind.

#### Covert transgressions

Covert transgressions did not appear to be deliberate acts of defiance but were expressions of autonomy. Children made their own choices about engaging in activities prohibited by parents that they may have suppressed in the parents’ presence but felt free to perform in the parents’ absence. In the home, children reported that they routinely transgressed against parental expectations about eating, personal behaviours, manners, and time spent on electronic devices, as well as the performance of responsibilities such as chores, homework, and music practice. One child reported breaking a snacking rule, “I snuck another ice cream cone and it was so delicious ‘cause it had chocolate chips and it was very, very, good!” (10-year-old male, Family 2). Another reported breaking a rule about sleep and video games, “Things that my parents don’t know about me today are that I play my Game Boy … a lot under my covers” (9-year-old male, Family 4).

Children’s reports of covert transgressions were also interesting for documenting potential changes in children’s autonomy motivation. Some children expressed signs of worry or guilt despite breaking transgressions. Other children were concerned about external repercussions for themselves or their parents if their transgressions were found out. For example, one child worried about the potential consequences of eating an extra piece of cake, “I am a little worried because if they find out I probably won’t get dessert tomorrow or anything …” (13-year-old male, Family 11). Another child discussed potential parent reactions while reporting a transgression about video games:

I didn’t tell my parents that I played a lot of video games …. I shouldn’t have played a lot of video games cause it’s not good. I should have just slept …. I didn’t tell them because I didn’t want them to get mad at me. (10-year-old male, Family 33)

Despite evidence of fretting about parental reactions, there was evidence that children made their own decisions about right and wrong. Some children appeared not to be concerned by parental disapproval or punishment. For example, a 13-year-old said the following about purchasing a caffeinated drink: “I bought a coffee drink on the way to school … It has caffeine in it though, so maybe my mom would have gotten mad at me” (Family 19). A 13-year-old girl stated the following in response to the expectation of not drawing pictures on her skin:

I gave him [brother] my tattoo pen thing and he drew like this big rose on my arm but my mum doesn’t like us drawing stuff on our arm because she says that it looks dirty …. I shouldn’t have let my brother draw on my arm because she, I know that she thinks it makes us look dirty. And I was just like I don’t really care. (Family 14)

#### Cognitive non-acceptance

Cognitive forms of resistance were identified from children’s reports of requests with which they may have complied behaviourally but did not accept internally. Cognitive resistance appeared to start with internal self-talk or private dialogues. Children expressed to themselves that they did not want to engage in the requested activity or had different reasons for not engaging in the activity. These expressions of resistance were experienced as internal affirmations of opposition towards parental demands or requests that children were compelled to obey or as negative evaluations of parental requirements. Cognitive non-acceptance is represented in the two subthemes of *conflicting goals* and *conflicting perspectives.*

#### Conflicting goals

The majority of participants in the study described incidents where they did not want to do what was requested of them. Children expressed resistance towards requests to complete chores, daily routines and other requests or commands that conflicted with their interests. Most of the children reported that they did not want to do daily chores or undertake household responsibilities. Examples included cleaning their room or the family home: “I didn’t want to sort my clothes” (13-year-old female, Family 23); practising musical instruments: “What I didn’t want to do was practise my piano” (13-year-old female, Family 16); completing homework: “My parents told me to do my homework and I did not really want to do that” (9-year-old male, Family 37); or multiple requests that made up their daily routines: “My mom asked me to take a shower … get ready for basketball practice … get out of bed … I did not want to not do any of those” (11-year-old female, Family 28). Although some internal dialogues were merely statements of preference for doing something other than what the parent had in mind for them, some children articulated reasoned explanations for their unhappiness with a parental request: “… I guess I didn’t want to do it because … I had been on my feet all day and I just wanted to sort of sit down” (13-year-old female, Family 17).

#### Conflicting perspectives

Children also reported not accepting the parental requirement or rule when they disagreed with parents on how to interpret a perspective. Children’s perspectives differed on a variety of topics, including how to interact with their siblings, rules about safety, having friends over, how to act in public, ideas about food, requests to put items away, and how chores are assigned or completed. A 13-year-old female expressed a difference of opinion over a computer rule:

I went on the computer without asking her which she doesn’t usually like it when I do that … I always do it anyways just so I know my iPod is charged for the day right because I have to listen to my music every day. (13-year-old female, Family 14)

This child regarded her own perspective on how to keep her devices charged as more reasonable than the parents’ rigid rules about computer use.

Children experienced non-acceptance of parental wishes when they differed from their own preferences in the moment. For example, a 13-year-old male provided the following perspective about being involved in his parent’s garage sale:

What I didn’t want to do was probably cleaning up for the garage sale. I didn’t want to do it because it was boring and repetitive, and it stopped me from doing other things that I would have rather been doing. (Family 6)

Children sometimes described parental requests that differed from their own point of view or needs as “unfair”. For example, a 9-year-old female explained how her understanding of appropriate behaviour for children differed from that of her mother:

My parent told me not to … play on stuff that isn’t yours because when we went shopping I played on stuff in the stores that I shouldn’t have. The reason they told me … that is because it’s not mine and I could break it and my mom would have to pay for it. I felt unfair about it because kids are meant to climb on things and I think everything should be meant to climb on. (Family 41)

## Discussion

This study addressed a gap in research on children’s strategies for resisting their parents’ attempts to control them in the developmental interval between early childhood and adolescence. Empirically, the findings provide, for the first time, a fine-grained description of children’s repertories of strategies for expressing resistance in middle childhood, and also provide clues about underlying developmental trends regarding children’s growing self-assertion, social skills, and mode of expression. Theoretically, the study supports an argument that children’s resistance to parental expectations reflects a normative process of developing autonomy in a relational context (Kuczynski & Hildebrandt, 1997). Practically, its focus on children’s expression of agency through resistance suggests new directions for considering the positive and negative implications of these behaviours for children’s health and well-being.

Children’s overt resistance strategies show continuities between earlier and later developmental periods. The assertive strategies reported by our middle childhood participants, including negotiation, argument, and expressive resistance, have close parallels in form and function to similar strategies found in early childhood (Kuczynski & Kochanska, 1990; Kuczynski et al., 1987) and adolescence (Parkin & Kuczynski, 2012). A noteworthy finding was that children in middle childhood frequently reported that they resisted some aspect of parental expectations by verbally negotiating with their parents. Negotiation, which includes strategies such as providing persuasive rationales for not complying, asking parents to justify their requests, and offers of compromises, is a skilful form of resistance from an interpersonal standpoint. By using negotiation, children not only assertively communicate their disagreement with some aspect of parental expectations but also engage with their parents relationally by considering their perspectives while pursuing their own autonomous goals (Kuczynski & Hildebrandt, 1997).

The study also shed light on children’s use of non-confrontational strategies. Counterparts to the two unassertive strategies, deflecting parental requests and minimal compliance, have been found in early childhood and adolescence. In research with preschool children, Kuczynski et al. (1987) and Kuczynski and Kochanska (1990) conceptualized passive non-compliance (ignoring or not acknowledging a request) as unassertive, aversive, and unskilful from the standpoint of an interpersonal influence strategy. However, it can be argued that the strategy of minimal compliance and the strategy of deflecting parental requests can be considered to be socially skilful strategies for expressing resistance. In a conversational analysis of young children’s responses to parental requests during mealtime, Kent (2012) described “incipient compliance”, a microlevel counterpart to the non-confrontational strategies in the present study. Kent argued that by making carefully timed preparatory steps towards compliance without actually following through, children can maintain a sense of autonomy over their own conduct without provoking a more forceful escalation from their parents. The phenomenon of non-confrontational strategies where children skilfully deflect compliance or resist by complying in a partial or ambiguous manner so as to resist control without provoking conflict is a potentially rich area of future research that greatly complicates traditional binary views of compliance and non-compliance.

Despite some continuities, it is possible to discern qualitative differences between the middle childhood narratives of resistance in the present study and the narratives of 13–19-year-old adolescents reported by Parkin and Kuczynski (2012). What stands out is the more confident nature of the adolescent expressions of resistance. Parkin and Kuczynski (2012, p. 650) observed that “their frank description of their strategies suggest[s] that they are more confident in their self assertion and more aware of their increased power in the parent–child relationship than has been noted at earlier periods”. In contrast, we characterize children in our middle childhood sample as “flirting with resistance”. Although children reported that they confronted parental authority by negotiation, arguing, and expressing opposition, their confrontations were more tentative and they showed greater readiness to back down than was evident in the Parkin and Kuczynski (2012) study. Rather than confidently asserting their opposition to rules and requests with which they disagreed, children in middle childhood often appeared to be exploring the boundaries of what their relationships with parents afforded them and the leeway for the expression of agency that they dared to exploit.

An important finding that distinguishes children’s resistance from that of young preschool children is that, in middle childhood as in adolescence (Parkin & Kuczynski, 2012), opposition is frequently expressed in covert and symbolic ways. This was found in the categories of covert transgressions and cognitive non-acceptance. In an older study (Kuczynski & Hildebrandt, 1997), children’s resistance to deviation in the parents’ absence was considered to be a marker of internal control. Consistent with this account of conscience development, some children acknowledged worrying about having their transgressions discovered. However, many children also reported that they did not particularly care about discovery or consider it when they transgressed. This child-as-agent perspective suggests that covert transgressions are more complex than traditional explanations referring to concepts such as failure of conscience or failure of parental discipline.

We suggest that children may choose to transgress against parental rules or expectations, not to defy their parents, but to freely pursue their own goals as they see fit when not constrained by the possibility of parental reprimand or merely the hassle of experiencing the disapproving gaze of their parents. In this agentic view, children may ignore parental messages or take them into consideration as they begin to make their own decisions and take responsibility for their own actions. A further development noted in the Parkin and Kuczynski (2012) study is that children may eventually engage in risky behaviours or transgress against parental values but do so in a way that does not imperil their valued relationships with their parents.

Another novel finding concerns cognitive resistance. The majority of children reported routine instances where they cognitively did not accept various parental messages, did not want to perform what was asked of them, or negatively evaluated the non-voluntary nature of their compliance. Cognitive resistance has previously been identified as a manifestation of human agency in oppressive circumstances (Goffman, 1961; Scott, 1990) as well as in clinical observations of clients who have endured abuse by family members or intimate partners (Wade, 1997). By engaging in resistance, cognitively oppressed individuals find small, concealed ways to control aspects of their experience and protect a sense of personal autonomy, even when they do not have personal control over their circumstances. Acts of cognitive resistance reported by our sample of children, even though they are more mundane, may have a similar function. Expressing non-acceptance of requests perceived as externally imposed may be meaningful for children as a way of restoring a sense of agency and may be associated with a sense of personal mastery.

Cognitive resistance is also relevant theoretically to the process of children’s internalization of values. According to Grusec and Goodnow (1994), in order for parental messages to be internalized, children must not only accurately perceive the content of the message but also motivationally accept the message. Previous research examined children’s evaluations of the appropriateness of parental discipline and practices for enforcing compliance with parental demands (Helwig, To, Wang, Liu, & Yang, 2014; Padilla-Walker & Carlo, 2004) and drew implications for children’s internalization of values. The present study suggests that future research should also consider children’s own evaluations of the requests. Most children in the current sample appeared to know what was expected of them and may have been compelled to comply. However, children’s narratives that they did not accept the appropriateness, fairness, or logic of parental expectations suggest that they also did not internalize the underlying messages that the parents intended.

The findings have implications for the health and well-being of children and parents. In behaviour-management approaches to family intervention and parental education (e.g., Forehand & McMahon, 2005), children’s non-conforming responses to parental control are conceptualized as “non-compliance”; failure to comply immediately, completely, and without compliance is considered to be coercive and problematic. An implication of the present study is that much of what has traditionally been labelled as non-compliance could alternatively be viewed as resistance, a normative expression of autonomy. As indicated by Robson and Kuczynski (this journal, 2018), even in clinical samples, it may be possible to distinguish developmentally normal forms of resistance from dysregulated aggression.

Moreover, research suggests that only unskilful or oppositional forms of resistance are related to negative outcomes in early childhood (Crockenberg & Litman, 1990; Kuczynski & Kochanska, 1990; Kuczynski et al., 1987; Van Petegem et al., 2015). Competent, assertive behaviour allows children to control elements of their environment that impact their daily lives (Dix, Stewart, Gershoff, & Day, 2007). In addition, there is evidence that children’s resistance is not necessarily associated with parental powerlessness or incompetence. Morrissey and Gondoli (2012) found that mothers with young adolescents who engaged in mature assertive but well-regulated non-compliance tended to have positive perceptions of their influence on their adolescents’ behaviour and granted their adolescents more input in decision making.

A nuanced view of children’s resistance that recognizes that positive or negative associations with well-being depend not on the fact of children’s resistance but on the way that children express resistance, has implications for parental education. We suggest that parents could be coached to understand that children’s resistance may have a positive role in their development of autonomy and also in their development of social skills. Parents may strategically wish to support their children’s emerging assertiveness when there is room for give and take. Parents may also wish to look for teachable moments and promote their children’s skills for achieving their goals by expressing opposition in a socially appropriate manner. Resistance in childhood is a sign of human agency and is an expected appearance in development. Resistance should be guided as a form of social competence, not suppressed and driven underground.

Several limitations need to be acknowledged in this study. The sample predominantly consisted of children of well-educated mothers, with little diversity in socioeconomic status or cultural diversity. It must be considered that the children in this sample may represent parent–child relationships where the culture and family climate afford leeway for children’s development and expression of autonomy through resistance. Thus, it is possible that the findings may not generalize to families that differ in educational levels or socio-economic status, or to collectivistic cultures that value obedience to authority and hierarchical power relations between parents and children (Trommsdorff & Kornadt, 2003). Nevertheless, children’s expression of autonomy by resistance have been found in collectivistic cultures such as China (Goh & Kuczynski, 2009) and Jamaica (Burke & Kuczynski, 2018), although the form that resistance takes may depend on the cultural context of parent–child relationships.

The narratives of children resisting parents in this study raise the important point of whose perspective one takes when describing children’s resistance. The idea that children are passive, parents are active, and parents are the legitimate authorities, is embedded not only in traditional models of socialization but also in culture and language (Kuczynski, Lollis, & Koguchi, 2003). Terms such as “non-compliance”, “coerciveness”, “disobedience”, “manipulative behaviour”, “misbehaviour”, “disrespect”, “sulking”, and “backtalk” can be found in the language of parental education, parental interventions, and everyday parenting. Such delegitimizing terms represent an adult perspective on children’s behaviour, which can only be applied from a position of greater power. Although we strove to represent children’s own accounts of resistance from their own perspective using descriptive language that was less evaluative, we are aware that theory is underdeveloped to fully understand the implications of children’s views of themselves as agents engaged in ordinary resistance. What can be said is that children described their experiences of resistance in an embodied way that included meaning making, moral indignation, views of themselves in relation to others, powerlessness, efficacy, and, sometimes, strong emotions of anger, exaltation, or despair. The children’s experiences of ordinary resistance were clearly meaningful to the children themselves. However, because children’s perspectives have not been generally recognized as legitimate experiences, making scientific sense of these experiences is a direction for future research and applied knowledge translation.
